# Extensive Orthodontic Movement in an Adolescent Patient With a Traumatized and Endodontically Treated Maxillary Central Incisor: Case Report With 7 Years of Follow‐Up

**DOI:** 10.1155/crid/5567947

**Published:** 2025-12-19

**Authors:** Elisa Souza Camargo, Layza Rossatto Oppitz, Ana Carolina Mastriani Arantes, Caio Seiti Miyoshi, Carlos Roberto Novakowski Filho, Oscar Mario Antelo Ribera, Patricia Kern Di Scala Andreis

**Affiliations:** ^1^ School of Medicine and Life Sciences, Pontifícia Universidade Católica do Paraná, Curitiba, Brazil, pucpr.br

**Keywords:** orthodontic appliance, orthodontics, tooth extraction, tooth movement, trauma

## Abstract

This article describes the orthodontic treatment involving a traumatized, endodontically treated upper central incisor that underwent extensive orthodontic movement. A male patient, 13 years and 1 month old, presented with the following conditions: mesofacial, convex profile; Class II skeletal pattern; angle Class I malocclusion; crowding of lower incisors; biprotrusion; and endodontic and restorative treatment of the traumatized left maxillary central incisor. Four first premolars were extracted to reduce dental protrusion and correct crowding of the lower incisors. Low‐magnitude forces were applied to the traumatized incisor and the movement time of this tooth was shorter than in the other teeth. Dental alignment and leveling, adequate overjet and overbite, and good positioning of the teeth in their bone bases were obtained. Orthodontic treatment with extensive movement of a traumatized, endodontically treated tooth was performed successfully and showed no root resorption 7.3 years postretention.

## 1. Introduction

The prevalence of dental trauma in incisors is 21%–33% in patients between 6 and 50 years of age [1, 2] with life‐long consequences for affected individuals [3]. The most heavily affected teeth are the upper central incisors, corresponding to 74.6%, insofar as playing is the greatest cause of trauma (64.2%), followed by sports (17.4%) [4]. Dental trauma may cause teeth to be avulsed, present a crown or root fracture, and promote pulpal necrosis or ankylosis [3, 5, 6], requiring endodontic treatment in these situations [7].

Apical root resorption is common in orthodontic treatment and is usually a consequence that coexists peacefully with orthodontics [8]. However, when a traumatized and endodontically treated tooth is submitted to orthodontic forces, the magnitude of root resorption is unpredictable and requires attention [9]. In addition, tooth ankylosis may occur during the movement of these teeth [10].

External apical root resorption consists of permanent loss of cementum from the apex of the root [8], and ankylosis consists of necrosis and disappearance of the periodontal ligament and consequent close contact of the alveolar bone with the cement. Both pathologies may hinder or prevent orthodontic treatment, or even lead to loss of the affected tooth [11]. Accordingly, the treatment objectives and the mechanics employed must be carefully planned [12].

The purpose of this paper is to present a clinical case report involving a traumatized, endodontically treated upper central incisor that underwent extensive orthodontic movement without developing apical root resorption during and after orthodontic treatment.

## 2. Case Report

This case report was performed in accordance with the ethical standards as laid down in the 1964 Declaration of Helsinki and its later amendments. The patient′s parents signed the informed consent form allowing the exposure of all treatment for didactic purposes.

A male patient aged 13 years and 1 month, with complete permanent dentition, was referred by a pediatric dentist for orthodontic evaluation. The patient′s main complaint was crowding of the lower incisors and dissatisfaction with facial profile. The left maxillary central incisor underwent dental trauma, with a fracture in the middle third of the crown. In the first pulp vitality assessment, the results were inconclusive, and after 15 days, during endodontic preservation, pulp necrosis was found and endodontic treatment was performed, followed by restorative treatment with the bonding of the tooth fragment. Despite the difference in shade between this tooth and the adjacent teeth, the patient and his legal guardians reported aesthetic satisfaction and did not request further interventions such as teeth whitening and direct or indirect veneers [13].

### 2.1. Assessment

**Figure 1:**
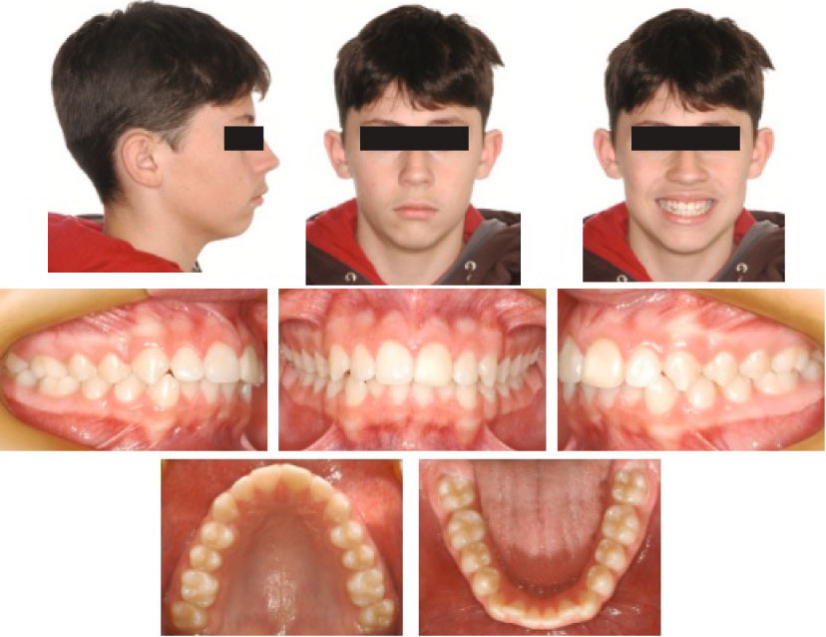
Pretreatment extraoral and intraoral photographs.

**Figure 2:**
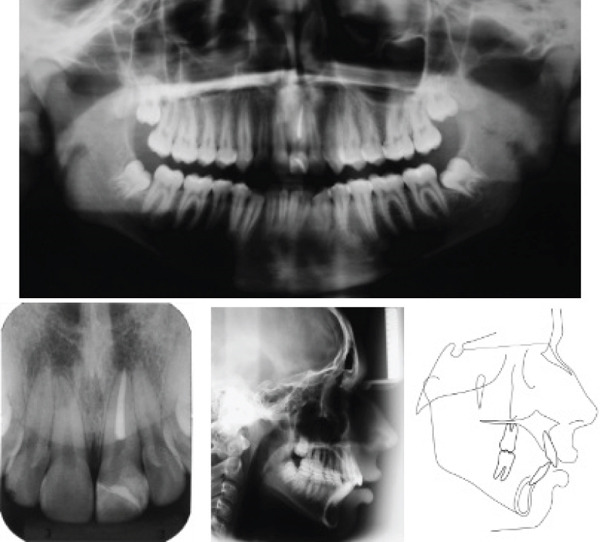
Pretreatment panoramic, periapical, and lateral cephalometric radiographs and tracing.

An extraoral examination revealed a symmetrical, mesofacial face; the profile was convex with labial sealing. An intraoral evaluation showed angle Class I malocclusion, 4‐mm overjet, 40% overbite, inferior crowding (−3.5 mm) and 1‐mm inferior midline deviation to the right (Figure 1). Cephalometric analysis (Figure 2 and Table 1) showed a Class II skeletal pattern (ANB = 8^°^) due to maxillary prognathism (SNA = 88^°^), with a harmonious facial growth pattern (GoGn: SN = 30^°^, FMA = 23^°^). The upper incisors were protruded (1 − NA = 7 mm) and retroclined (1 : NA = 18^°^), and the lower incisors were protruded (1 − NB = 11 mm) and proclined (1 : NB = 39^°^, IMPA = 106^°^).

**Table 1:** Cephalometric measurements at pretreatment and posttreatment.

**Measurements**	**Normal**	**Pretreatment**	**Posttreatment**
Skeletal pattern			
SNA (°)	82	86.5	85
SNB (°)	80	79.5	80.5
ANB (°)	2.0	7	4.5
SN‐GoGn (°)	32	32	31
*y*‐axis (°)	59	58.5	60
Facial angle (°)	87	90.5	89.5
Convexity (°)	0	9	9
Ao‐Bo (mm)	0	1	0
FMA (°)	25	24	23
Dental pattern			
1.NA (°)	22	20	15.5
1‐NA (mm)	4	4.5	2
1.NB (°)	25	36	31
1‐NB (mm)	4	10.5	6
Interincisal angle (°)	131	118	129
IMPA (°)	90	102	96
Profile			
*Z*‐angle (°)	75	61	72

The panoramic radiograph showed endodontic and restorative treatment of the traumatized left maxillary central incisor, and the third molars were in development. The periapical radiograph revealed intact dental roots, including that of the left maxillary central incisor (Figure 2).

The possibility of the traumatized incisor being ankylosed was ruled out since the trauma and endodontic treatment occurred 3 years before the start of orthodontic treatment, and clinically, there was no infraocclusion of the tooth. In addition, the percussion test revealed a clear and resonant sound, similar to that of the adjacent teeth, and the periapical radiograph showed integrity of the periodontal ligament [11].

### 2.2. Aims of Treatment

The objectives of the treatment were as follows: (1) to correct the dental biprotrusion; (2) to improve the profile convexity; (3) to obtain adequate dental alignment, overjet, and overbite; and (4) to correct the deviation of median lines.

There were the following treatment alternatives:

A. Alignment and leveling using an extrabuccal device as an anchor, and Class III elastics and stripping to obtain space in the lower arch to minimize dental projection.
B. Extraction of the four first premolars to reduce dental biprotrusion and improve facial profile, and also obtain space to allow dental alignment.
C. Combined orthodontic–orthognathic treatment to correct skeletal discrepancies and harmonize facial profile.

### 2.3. Treatment

**Figure 3:**
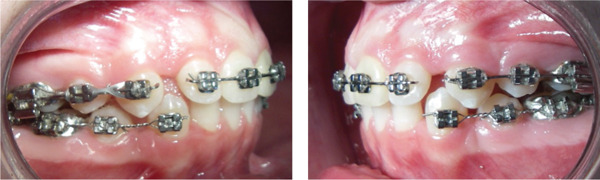
Intraoral photographs of the end of the canine retraction stage and the beginning of the alignment and leveling of the upper incisors.

The patient′s legal guardians consent was obtained, and they did not agree with the orthodontic‐surgical treatment option. Therefore, the second treatment option was chosen since it ensured more stable results in the sagittal position of the incisors, and an improvement in the facial profile. The first upper and lower premolars were extracted to reduce dental protrusion and correct crowding of lower incisors. A standard edgewise 0.022 in‐slot fixed device was used, together with an extrabuccal appliance to anchor the maxillary molars. The upper incisors were included in the device after alignment and leveling of the posterior teeth, and final stage of canine retraction (Figure 3), in order to minimize the movement time of the left maxillary central incisor, and consequently reduce the possibility of root resorption [14]. Although the total treatment time is similar in cases of mass retraction or two‐stage retraction, the time for alignment and leveling may be longer in cases of mass retraction [15], which is contraindicated in cases of traumatized teeth, as it may lead to a higher risk of root resorption [14].

Retraction arches were then placed with helical loops to retract the incisors, using low magnitude forces and activating every 45 days. Periapical radiographs of the upper and lower incisors were taken periodically to observe the dental roots, especially of the traumatized tooth.

**Figure 4:**
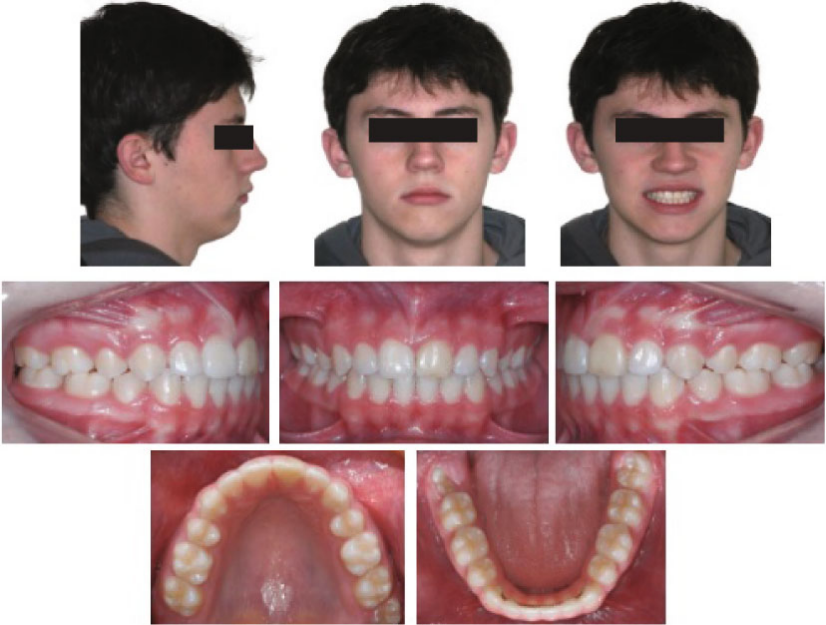
Posttreatment extraoral and intraoral photographs.

**Figure 5:**
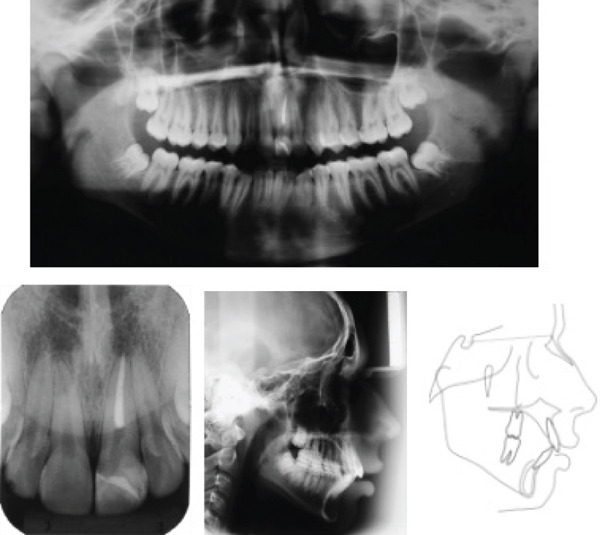
Posttreatment panoramic, periapical, and lateral cephalometric radiographs and tracing.

**Figure 6:**
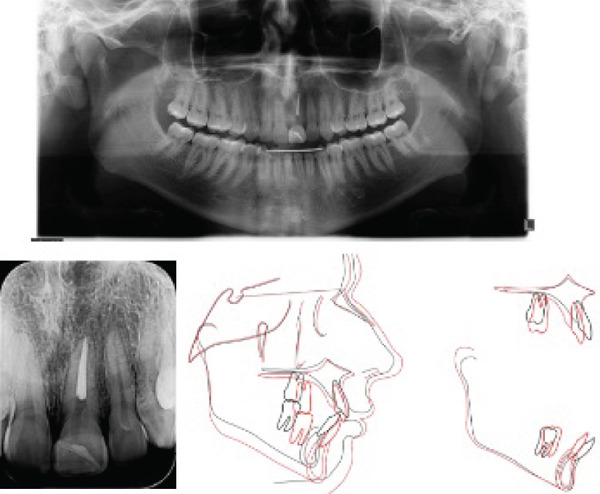
Panoramic and periapical radiographs at the 7 years and 3 months follow‐up and cephalometric superimposition (black, pretreatment; red, posttreatment).

After 2 years and 11 months of treatment the fixed apparatus was removed, and a superior wraparound retainer was installed. A fixed canine‐to‐canine retainer was bonded in the lower arch. Dental alignment and leveling, adequate overjet and overbite, and good positioning of the teeth in their bone bases were obtained. Deviation of the lower midline was corrected, and the facial profile was improved (Figure 4). The posttreatment panoramic and periapical radiographs showed healthy tissues, and no root resorption of the traumatized, endodontically treated left maxillary central incisor (Figure 5 and Table 1). The superimposition of the lateral cephalometric radiograph tracings shows the skeletal, dental, and soft tissue alterations that favored facial aesthetics and treatment stability (Figure 6).

**Figure 7:**
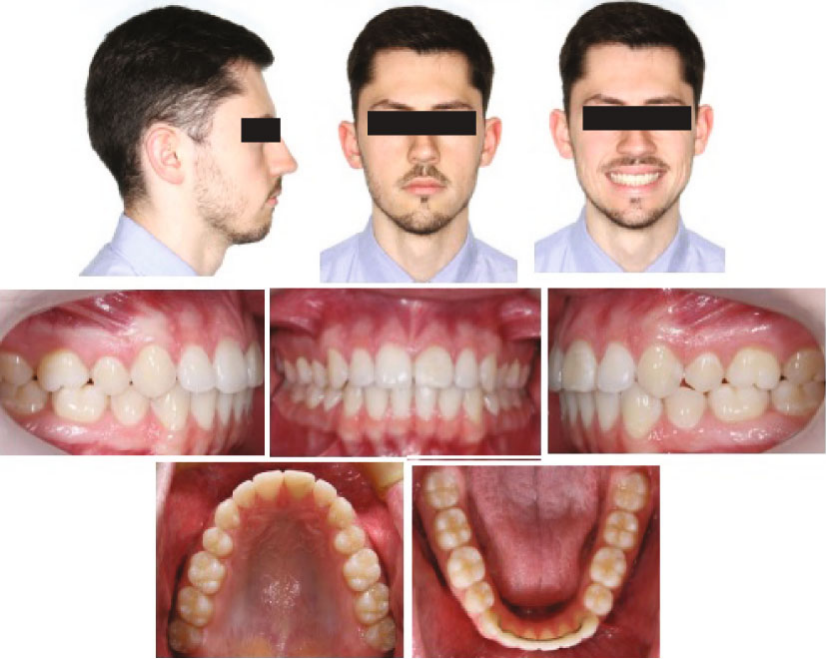
Extraoral and intraoral photographs at the 7 years and 3 months follow‐up.

Then, 7 years and 3 months after the end of treatment, the clinical results achieved were found to be stable (Figure 7), and the root of the traumatized central incisor showed no radiographic signs of root resorption (Figure 6).

## 3. Discussion

Clinical cases with Class II skeletal patterns and facial profile disharmony are strong candidates for more invasive approaches such as orthodontic‐surgical treatment [16]. Mandibular advancement surgery and mentoplasty or the combined maxillomandibular technique are options with aesthetic advantages. The outcomes improve aesthetics, occlusion, contribute to better long‐term results and enhanced patient satisfaction [17]. However, the high financial cost and risks inherent in the surgical procedure may make this choice unfeasible [16], as in the case reported here. Thus, the treatment option called for extraction of the premolars due to crowding and protrusion of the lower incisors and to aid in the camouflage of Class II skeletal pattern [16]. It was believed that if the premolars were not extracted, the lower incisors would project forward and risk stability of alignment [14]. This could cause periodontal resection [18] and would worsen, or, at best, not improve the already convex facial profile [19].

However, there was concern regarding the movement of the upper incisors, since the patient had suffered trauma to the central incisor 3 years before the beginning of the orthodontic treatment, with fracture in the middle third of the crown, but without pulp exposure. Considering that a traumatized tooth may develop inflammatory root resorption (external and internal) and pulp necrosis, as observed in this case report, endodontic treatment was indicated and performed. The risks of moving traumatized teeth treated endodontically involve not only root resorption, but also ankylosis and fractures, due to extensive restoration [10, 20].

In the clinical case described here, ankylosis of the traumatized tooth was ruled out by percussion, occlusion and radiographic examinations, and the presence of mobility [11]. However, if ankylosis was suspected, CBCT would be requested, as it is the most accurate method for diagnosing this condition [21]. The prudent clinical approach would be to plan the case without tooth extractions during the initial alignment and leveling phase until the movement of this tooth, induced by orthodontic mechanics, is confirmed. If ankylosis was confirmed, other treatment options could be indicated, such as surgical luxation or corticotomy of the ankylosed tooth, replacement with a dental implant with bone reconstruction, or osteogenic distraction [11].

Panoramic and periapical radiographs were taken during treatment planning to evaluate the condition of the dental tissues and other structures, according to the protocol suggested by Levander and Malmgren [22]. Control radiographs were done quarterly to detect the appearance of possible root resorption, considering that this procedure is recommended for not only traumatized, but also nontraumatized teeth.

The panoramic and periapical radiographs of the case described herein did not present images suggestive of apical root resorption in left maxillary central incisor at the end of treatment. Root resorption is usually characterized by apical rounding and is a common occurrence during orthodontic treatment [20, 23]. However, there is a conflict of evidence in the literature regarding whether endodontic treatment can be considered a risk factor for increased root resorption during orthodontic treatment. Some authors [24, 25] mention that traumatized teeth that have undergone canal treatment are associated with increased susceptibility to root resorption during orthodontic treatment, whereas others [25, 26] assert that the frequency of this pathology is reduced.

The integrity of the root apex observed after orthodontic movement of the traumatized incisor corroborates the findings reported by Lee and Lee [25] who found that teeth previously treated endodontically had less apical root resorption than teeth with vitality and that this resorption is influenced by age, duration of treatment, type of treatment, and periapical conditions. Similarly, Alhadainy et al. [26] concluded in a systematic review that endodontic treatment does not seem to increase external root resorption induced by orthodontic treatment. However, it is common among many clinicians to believe that endodontically treated teeth are more susceptible to apical root resorption, even though these teeth respond to orthodontic treatment similarly to vital teeth [14].

Root resorption is a complex process involving the interaction between various molecular signaling pathways that stimulate odontoclasts/cementoclasts to resorb cementum and dentin [9]. According to Brudvik and Rygh [27], a longer treatment time correlates with the presence of apical root resorption in healthy, non‐traumatic upper incisors. Because traumatized teeth are already more prone to root resorption [28], they should be included in the device as late as possible into the treatment. It is reported that reduced orthodontic movement time [14] and low‐magnitude forces [9] decreases the possibility of root damage. Based on this, care was taken in the treatment of the present clinical case to use light force, especially during activation of the incisor retraction loops. In addition, the brackets were bonded to the incisors in the final stage of canine retraction, thus reducing the movement time of the traumatized tooth.

Although it was verified at the end of the treatment that the root of the left maxillary central incisor was intact, clinical, and radiographic follow‐up continued to be performed by the orthodontist and endodontist, during periods of retention and postretention. It was observed that the radiographic image of the tooth suggested continued normality, and the occlusion was stable after 7 years and 3 months of removal of the device.

Notwithstanding the prognosis of trauma‐affected teeth is uncertain [23], orthodontists often must include them in their treatment plans. The success of orthodontic treatment depends on careful diagnosis and care during the biomechanics employed therein.

## 4. Conclusion

Orthodontic treatment, when there is extensive movement of traumatized and endodontically treated teeth, can be performed successfully, as long as the movement time of this tooth is reduced, and low‐magnitude forces are applied.

## Ethics Statement

The patient signed an informed consent form for the publication of his treatment, including all documents and data involved.

## Conflicts of Interest

The authors declare no conflicts of interest.

## Author Contributions

Elisa Souza Camargo: acquisition of data (patient treatment), made substantial contributions to conception and design, and involved in drafting the manuscript and revising it critically for important intellectual content. Layza Rossatto Oppitz: revising it critically for important intellectual content. Ana Carolina Mastriani Arantes: drafting the manuscript. Caio Seiti Miyoshi: acquisition of data (patient treatment). Carlos Roberto Novakowski Filho: acquisition of data (patient treatment). Oscar Mario Antelo Ribera: made substantial contributions to conception and design. Patricia Kern Di Scala Andreis: involved in drafting the manuscript and revising it critically for important intellectual content.

## Funding

The authors received no specific funding for this work.

## Data Availability

Data is available from the first author upon request.
